# Isosakuranetin ameliorates hypertension in rats induced by L-NAME

**DOI:** 10.1186/s40780-025-00529-z

**Published:** 2025-12-17

**Authors:** Rungusa Pantan, Ratchanaporn Chokchaisiri, Apichart Suksamrarn, Chainarong Tocharus

**Affiliations:** 1 Department of Anatomy, Faculty of Medicine, Chiang Mai University, Chiang Mai, 50200 Thailand; 2 Department of Chemistry, School of Science, University of Phayao, Phayao, 56000 Thailand; 3 Department of Chemistry and Center of Excellence for Innovation in Chemistry, Faculty of Science, Ramkhamhaeng University, Bangkok, 10240 Thailand

**Keywords:** Isosakuranetin, Flavanone, Hypertension, Anti-hypertensive, Oxidative stress

## Abstract

**Background:**

Hypertension is a major global health problem that often develops without noticeable symptoms. Current treatments and complementary approaches focus on improving endothelial function and enhancing nitric oxide (NO) production to promote vasodilation and lower blood pressure. Isosakuranetin, a flavanone found in *Chromolaena odorata* leaves, has shown potential antihypertensive properties. This study aimed to investigate the effects of isosakuranetin on L-N^G^-Nitroarginine methyl ester (L-NAME)–induced hypertension, focusing on its ability to enhance NO production and reduce oxidative stress.

**Methods:**

This study investigated the antihypertensive effects of the flavanone isosakuranetin in male Wistar rats (n = 8 per group). Hypertension was induced by L-NAME, a nitric oxide synthase inhibitor, for four weeks, followed by treatment with isosakuranetin (10, 20, or 40 mg/kg) or enalapril (10 mg/kg) for an additional two weeks. Systolic blood pressure (SBP), heart rate, and body weight were monitored weekly. After six weeks, the effects of isosakuranetin on NO level and oxidative stress were assessed using the Griess reaction, 2’,7’-dichlorofluorescein diacetate (DCF-DA), and superoxide dismutase (SOD) activity assays.

**Results:**

The results demonstrated that the SBP was significantly reduced in the isosakuranetin treatment group when compared to the hypertensive group. Additionally, isosakuranetin treatment significantly restored plasma nitrate/nitrite levels and showed the potential to reduced oxidative stress, as indicated by the decrease in reactive oxygen species (ROS) levels and a significant increase in SOD activity.

**Conclusion:**

These findings suggest that isosakuranetin is a promising natural compound for managing hypertension, demonstrating its potential for clinical application.

**Supplementary Information:**

The online version contains supplementary material available at 10.1186/s40780-025-00529-z.

## Background

Hypertension, one of the most common age-related disorders and a major risk factor for cardiovascular diseases, is considered a significant global health problem. Studies have reported that hypertension can damage cerebral blood vessels and is directly associated with the development of cognitive impairment, including dementia and Alzheimer’s disease [1–4].

Strategies for treating hypertension include lifestyle modification, medication, and collaborative care approaches. Additionally, supplements containing natural compounds are increasingly recognized for their potential in preventing and managing high blood pressure [5]. Various natural products exhibit antihypertensive effects through multiple mechanisms, including antioxidant and anti-inflammatory activities, regulation of the renin-angiotensin system, and improvement of endothelial function [6–10].

Chronic high blood pressure is associated with endothelial cell degeneration, leading to reduced production of vasodilators such as nitric oxide (NO), impaired vessel dilation, and increased blood pressure [11–13]. Additionally, NO helps prevent the adhesion of white blood cells, which initiates cytokine production and correlates with prolonged and excessive vascular wall inflammation, further activating the proliferation and migration of vascular smooth muscle cells [14–16]. These events contribute to the development of vascular diseases, including atherosclerosis and carotid artery disease [17, 18].

In studies of hypertension, suppression of endothelial nitric oxide synthase (eNOS) is commonly used to induce high blood pressure in animal models [19–22]. The nitric oxide synthase inhibitor L-N^G^-Nitroarginine methyl ester (L-NAME) is typically used to inhibit eNOS activity and reduce vascular NO production, leading to hypertension. Furthermore, chronic administration of L-NAME in rats raises blood pressure, accompanied by increased expression of inflammatory markers and elevated oxidative stress [23, 24]. The overproduction of reactive oxygen species (ROS) causes oxidative stress to exceed the dismutation capacity of superoxide dismutase (SOD), which is harmful to cells and further promotes inflammation [25]. These processes collectively contribute to vascular dysfunction and play a critical role in the pathophysiology of hypertension.

*Chromolaena odorata* (*C. odorata*), also known as *Eupatorium odoratum*, exhibits diverse medicinal properties, including analgesic, antibacterial, antioxidant, antimalarial, anti-inflammatory, antidiabetic, and wound-healing activities [26–29]. Previous studies have identified many flavones derived from *C. odorata* [30–32]. Isosakuranetin is one of the flavanones isolated from *C. odorata* and has been demonstrated to be a potent antioxidant [33]. Furthermore, it has been reported that isosakuranetin, isolated from Brazilian green propolis, demonstrates an antihypertensive effect in spontaneously hypertensive rats [34]. However, the role of isosakuranetin in ameliorating hypertension has not been clarified. In addition, there is currently no information regarding the effect of isosakuranetin on restoration of endothelial function in hypertension induced by L-NAME—a model that closely mimics essential hypertension in humans. Moreover, evidence relating to the modulation of the balance between ROS and SOD by isosakuranetin remains limited. Therefore, this study aims to elucidate the mechanisms by which isosakuranetin reduces oxidative stress and restores nitric oxide bioavailability in an L-NAME-induced hypertension model. This approach is crucial for evaluating the potential of this compound for clinical application and for developing natural products as alternative options for hypertension treatment in humans.

## Materials and methods

### Isolation and identification of Isosakuranetin

**Fig. 1:**
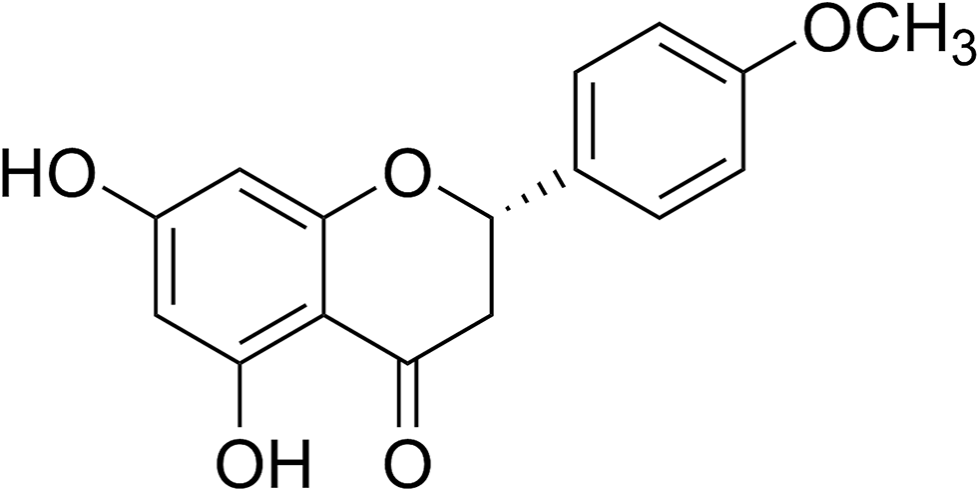
Chemical structure of isosakuranetin

Isosakuranetin was isolated from the same source of *C. odorata* using the method previously described by our group [35], with some modifications. Briefly, air-dried leaves of *C. odorata* (10 kg) were ground and sequentially extracted with *n*-hexane and ethyl acetate (EtOAc) at room temperature. The resulting extracts were filtered and concentrated to dryness under reduced pressure at 40–45 °C. The EtOAc extract, which contained isosakuranetin, was further fractionated using column chromatography with a gradient solvent system of *n*-hexane–EtOAc, pure EtOAc, and EtOAc–methanol (MeOH), employing increasing amounts of the more polar solvent. The eluates were analyzed by thin-layer chromatography (TLC), resulting in three groups of fractions. Group 2 was subjected to further purification with Sephadex LH-20 chromatography, eluting with 40% dichloromethane (CH_2_Cl_2_) in methanol, and yielded three sub-fractions. The first sub-fraction was repeatedly recrystallized from CH_2_Cl_2_ to obtain isosakuranetin (620 mg), as shown in Fig. 1. The structure of isosakuranetin was confirmed by comparison of its spectroscopic data with reference values reported in the literature [36]. The purity of the isolated isosakuranetin was determined to be greater than 95% by TLC and nuclear magnetic resonance (NMR) analysis.

### Animal groups and treatments

Male Wistar rats (150–200 g) were obtained from Nomura Siam International Co., Ltd. (Patumwan, Bangkok, Thailand). Animals were individually housed in standard cages under controlled conditions (12 h light/dark cycle; 25 ± 2 °C) with ad libitum access to food and water. Hypertension was induced in groups 3–7 by L-NAME at 40 mg/kg/day for 4 weeks, administered in the drinking water; water intake was recorded daily to verify dosing. Rats were then randomly allocated to seven groups (*n* = 8 per group; total *N* = 56): (1) normal control; (2) normal + isosakuranetin (40 mg/kg); (3) hypertensive control; (4–6) hypertensive + isosakuranetin (10, 20, or 40 mg/kg); and (7) hypertensive + enalapril (10 mg/kg). At the end of the study, rats were deeply anesthetized and euthanized with an overdose of pentobarbital sodium (150 mg/kg, i.p.); death was confirmed by cessation of heartbeat and respiratory arrest. After that, blood and thoracic aortae were collected immediately. Aortae were placed in ice‑cold Krebs–Henseleit buffer (in mM: NaCl 118, KCl 4.7, CaCl_2_ 2.5, MgSO_4_ 1.2, KH_2_PO_4_ 1.2, NaHCO_3_ 25, glucose 11; pH 7.4) for subsequent analyses.

### Effect of Isosakuranetin on blood pressure, heart rate, and body weight

Following 4 weeks of L-NAME induction, hypertensive rats received isosakuranetin (10, 20, or 40 mg/kg) or enalapril (10 mg/kg) for an additional 2 weeks to evaluate the effects of isosakuranetin on blood pressure. Systolic blood pressure was measured weekly using the tail‑cuff method with a noninvasive system (model MK‑1030; Muromachi Kikai Co., Ltd., Kyoto, Japan). Heart rate and body weight were recorded weekly.

### Effect of Isosakuranetin on NO production

Plasma NO levels were indirectly quantified by measuring nitrite (NO_2_^-^) using the Griess reagent, following the method of Green et al. (32). Blood was collected by cardiac puncture into EDTA-coated tubes and centrifuged at 2,000 × g for 10 min at 4 °C to obtain plasma. Then, plasma was deproteinized via ultrafiltration using centrifugal concentrators. The supernatants were then reacted with 1% sulfanilamide in 5% phosphoric acid, followed by the addition of 0.1% N-(1-naphthyl) ethylenediamine dihydrochloride. Absorbance readings were taken at 540 nm using a microplate reader (BioTek Instruments, Winooski, VT, USA). Nitrite concentrations in the samples were calculated by comparison to a sodium nitrite (NaNO_2_) standard curve.

### Effect of Isosakuranetin on the production of ROS

To assess the antioxidative effect, ROS levels were quantified using the fluorescent probe 2′,7′-dichlorodihydrofluorescein diacetate (DCFH-DA). The aortic tissue was first homogenized and then incubated with 1 µM DCFH-DA at 37 °C for 30 min in the dark to enable cellular uptake and enzymatic conversion to non-fluorescent DCFH. In the presence of ROS, DCFH is rapidly oxidized to the highly fluorescent 2′,7′-dichlorofluorescein (DCF). Following incubation, fluorescence intensity was measured using a microplate reader (DTX800, Beckman Coulter, Austria) with excitation and emission wavelengths set at 485 nm and 535 nm, respectively.

### Effect of Isosakuranetin on SOD activity

The activity of SOD, a critical antioxidant enzyme, was evaluated using the Superoxide Dismutase Assay Kit (Cayman Chemical Company, Ann Arbor, MI, USA) to detect the dismutation of superoxide anion radicals through the xanthine oxidase–hypoxanthine system. Briefly, isolated aortic rings were homogenized, and the supernatant was collected for assay. Total protein concentration of each sample was quantified by the Bradford assay, and SOD activity was measured according to the kit protocol. Enzymatic activity was calculated as U per mg protein. Absorbance was read immediately at 450 nm using a microplate reader (BioTek Instruments, Winooski, VT, USA).

### Statistical analysis

Results are expressed as mean ± SEM. Statistical significance was set at *p* < 0.05.Data were analyzed using GraphPad Prism 9.0. One-way ANOVA was used to compare among treatment groups, followed by Dunnett’s post-hoc test for multiple comparisons with control group. Two-way repeated-measures ANOVA was used to analyze SBP over time and treatment, followed by Tukey’s HSD post-hoc test.

## Results

### Effect of Isosakuranetin on blood pressure, heart rate, and body weight

**Fig. 2:**
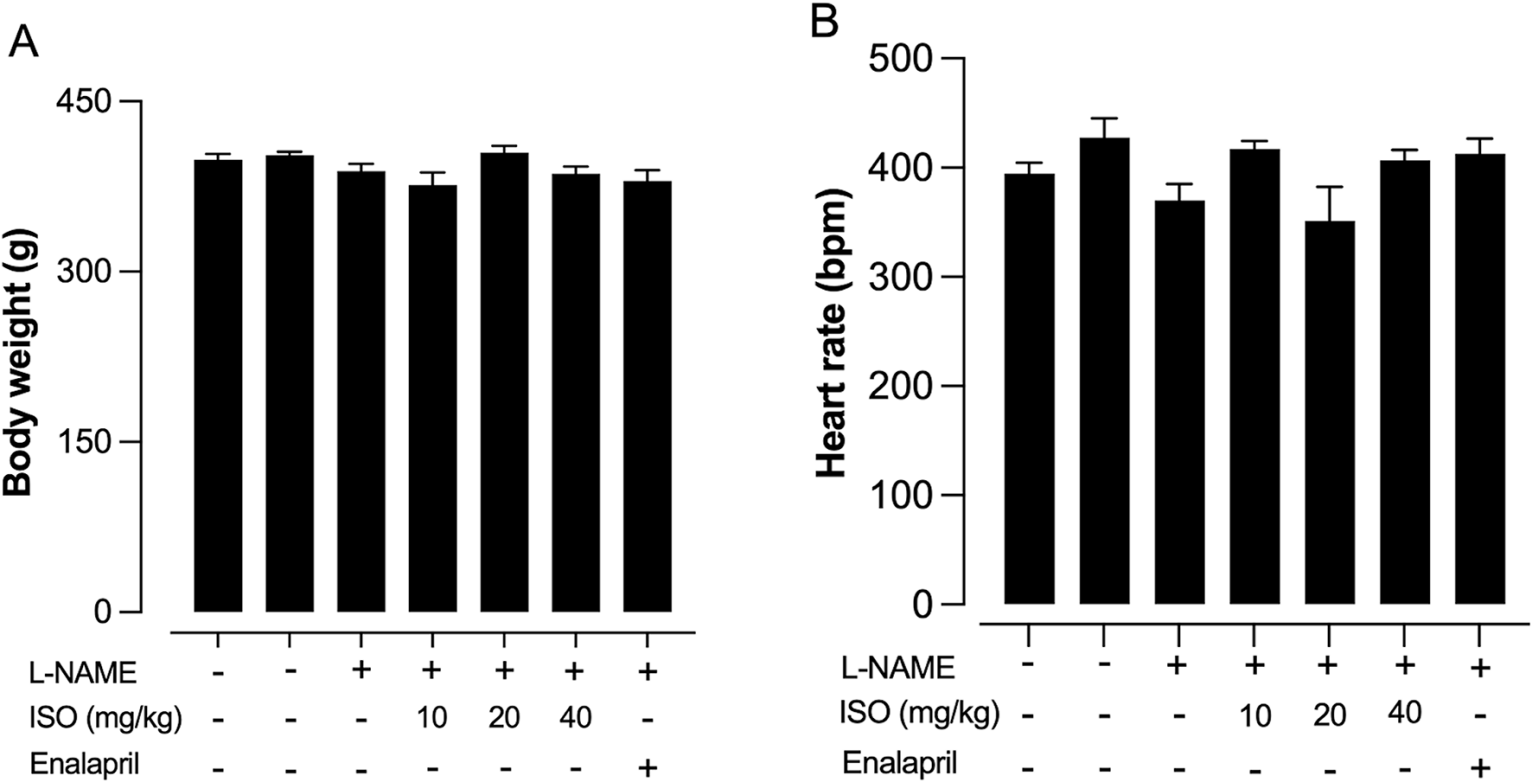
Effect of isosakuranetin (ISO) on body weight (**A**) and heart rate (**B**) in rats with L-NAME-induced hypertension. Rats were treated with L-NAME to induce hypertension and subsequently administered ISO at doses of 10, 20, and 40 mg/kg. Enalapril 10 mg/kg was used as a positive control. Bars represent the mean ± SEM for each group. No statistically significant differences were observed among all groups

After receiving L-NAME, the SBP gradually increased over 4 weeks, showing a marked difference compared to the control group. After 4 weeks, the animals continued to receive L-NAME, coupled with isosakuranetin at various concentrations (10, 20, 40 mg/kg), or enalapril at 10 mg/kg. The results revealed that isosakuranetin treatment decreased the SBP in a dose-dependent manner, similarly to the enalapril-treated group, as shown in Table 1. At the end of the 6th week, the SBP of hypertensive rats was significantly increased compared to the control group (*p* < 0.001). However, the SBP of rats treated with isosakuranetin (10, 20, and 40 mg/kg) was significantly decreased with a *p*-value less than 0.01 and 0.001 when compared to hypertension group. Consistent with the SBP of rats treated with enalapril, which was reduced significantly (*p* < 0.001) compared to hypertension group. Interestingly, treatment with isosakuranetin alone did not alter SBP compared to the control group. Additionally, all treatments did not affect heart rate or body weight in any group as showed in Fig. 2A and B.

**Table 1:** Systolic blood pressure (SBP) in control and experimental groups treated with ISO or Enalapril over 6 weeks

Group	Control	ISO (40)	HT	HT + ISO (10)	HT + ISO (20)	HT + ISO (40)	HT + Enalapril
Basal	122.8 ± 1.6	122.2 ± 1.1	124.8 ± 1.2	123.1 ± 1.1	122.4 ± 1.4	125.7 ± 1.6	122.9 ± 1.4
Week 1	121.1 ± 1.3	120.4 ± 2.6	124.4 ± 3.4	125.8 ± 4.3	123.6 ± 4.1	126.1 ± 2.2	119.9 ± 5.2
Week 2	122.9 ± 1.6	122.5 ± 1.5	143.8 ± 4.7	137.1 ± 3.0*	137.0 ± 5.4	135.5 ± 4.5	141.4 ± 5.2
Week 3	122.5 ± 1.9	119.5 ± 2.8	154.5 ± 4.4**	153.9 ± 4.8**	156.4 ± 4.8**	150.8 ± 3.4**	152.2 ± 5.0**
Week 4	122.5 ± 1.6	123.1 ± 1.5	169.0 ± 1.4***	170.1 ± 5.0***	167.9 ± 1.7***	167.9 ± 1.5***	171.4 ± 1.2***
Week 5	123.2 ± 1.4	122.4 ± 3.4	177.4 ± 2.3	172.0 ± 4.3	161.6 ± 2.9^#^	158.5 ± 1.9^###^	157.8 ± 3.1^##^
Week 6	124.7 ± 1.9	120.0 ± 2.5	186.8 ± 2.6	164.6 ± 3.4^##^	154.5 ± 2.5^###^	151.9 ± 2.4^###^	141.0 ± 2.8^###^

Values are expressed as mean  ±  SEM, Two-way repeated-measures ANOVA with Tukey’s post hoc test

SBP = Systolic Blood Pressure; ISO = Isosakuranetin (dose in mg/kg); HT = Hypertensive group

*p < 0.05, **p < 0.01, ***p < 0.001 compared to the control group

^#^*p* < 0.05, ^##^*p* < 0.01, ^###^*p* < 0.001 compared to the HT group

### Plasma nitrate/nitrite

**Fig. 3:**
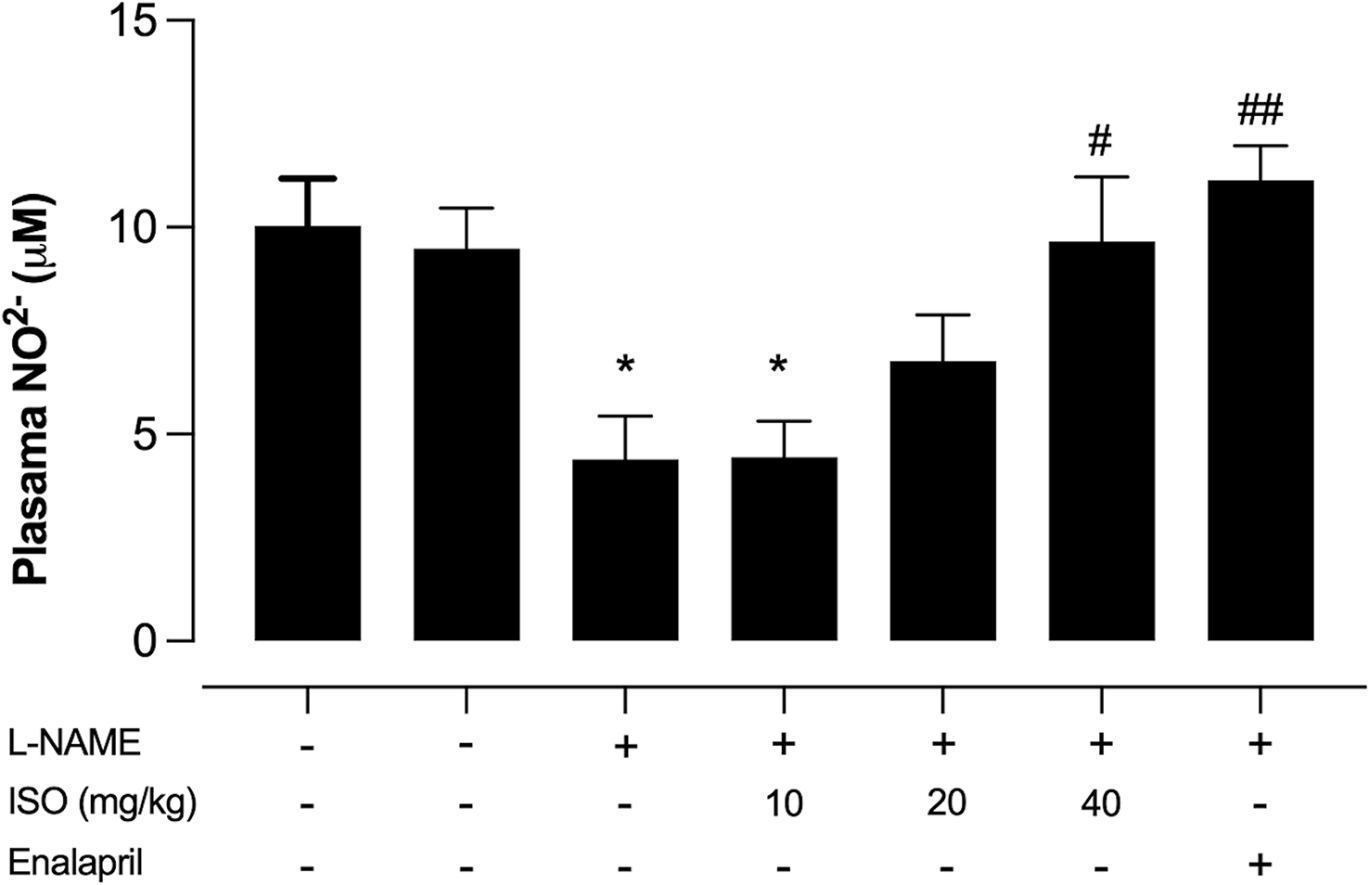
Effect of isosakuranetin (ISO) on nitric oxide level. The alteration was demonstrated as plasma nitrite (NO^2-^) level which measured in the control group, control group treated with ISO 40 mg/kg, hypertension group (L-NAME treated group), and hypertension groups treated with ISO at 10, 20, or 40 mg/kg or enalapril at 10 mg/kg. Data are presented as mean ± SEM. *p < 0.05 vs. control group; ^#^*p* < 0.05 and ^##^*p* < 0.01 vs. hypertension group

To evaluate the level of NO in plasma, NO_2_^-^, the oxidized product of NO, was measured using the Griess reaction assay. In the hypertension group, the level of NO_2_^-^ was significantly diminished after receiving L-NAME compared to the control group (*p* < 0.05). However, the level of NO^2-^ considerably increased following treatment with isosakuranetin and enalapril. The results revealed that the level of NO^2-^ was significantly increased in rats treated with 40 mg/kg of isosakuranetin (*p* < 0.05) and 10 mg/kg of enalapril (*p* < 0.01) as showed in Fig. 3.

### ROS

**Fig. 4:**
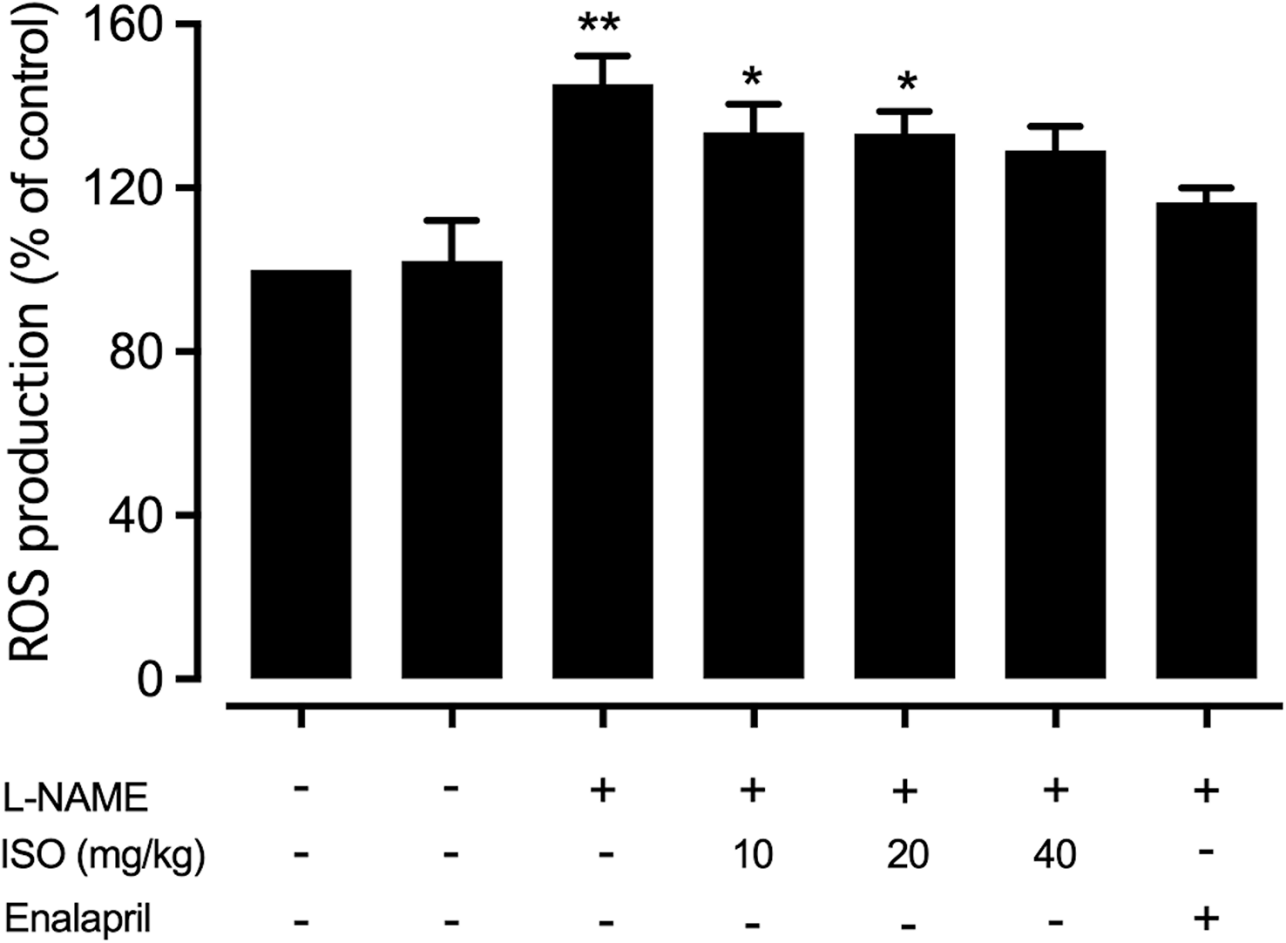
Effect of isosakuranetin (ISO) on reactive oxygen species (ROS) production. The percentage of ROS levels compared to the control group was measured in different groups: control group treated with ISO 40 mg/kg, hypertension group (L-NAME treated group), and hypertension groups treated with ISO at 10, 20, or 40 mg/kg or enalapril at 10 mg/kg. Data are presented as mean ± SEM. Statistical significance is indicated as *p < 0.05 and **p < 0.01 compared to the control group

The level of ROS production was measured using the DCF-DA assay and the data were analyzed as a percentage of the control. The results demonstrated that L-NAME significantly increased ROS production compared to the control group (*p* < 0.01). After treatment with isosakuranetin or enalapril, the level of ROS production was notably decreased; however, the reduction was not statistically significant compared to the hypertension group as showed in Fig. 4.

### SOD

**Fig. 5:**
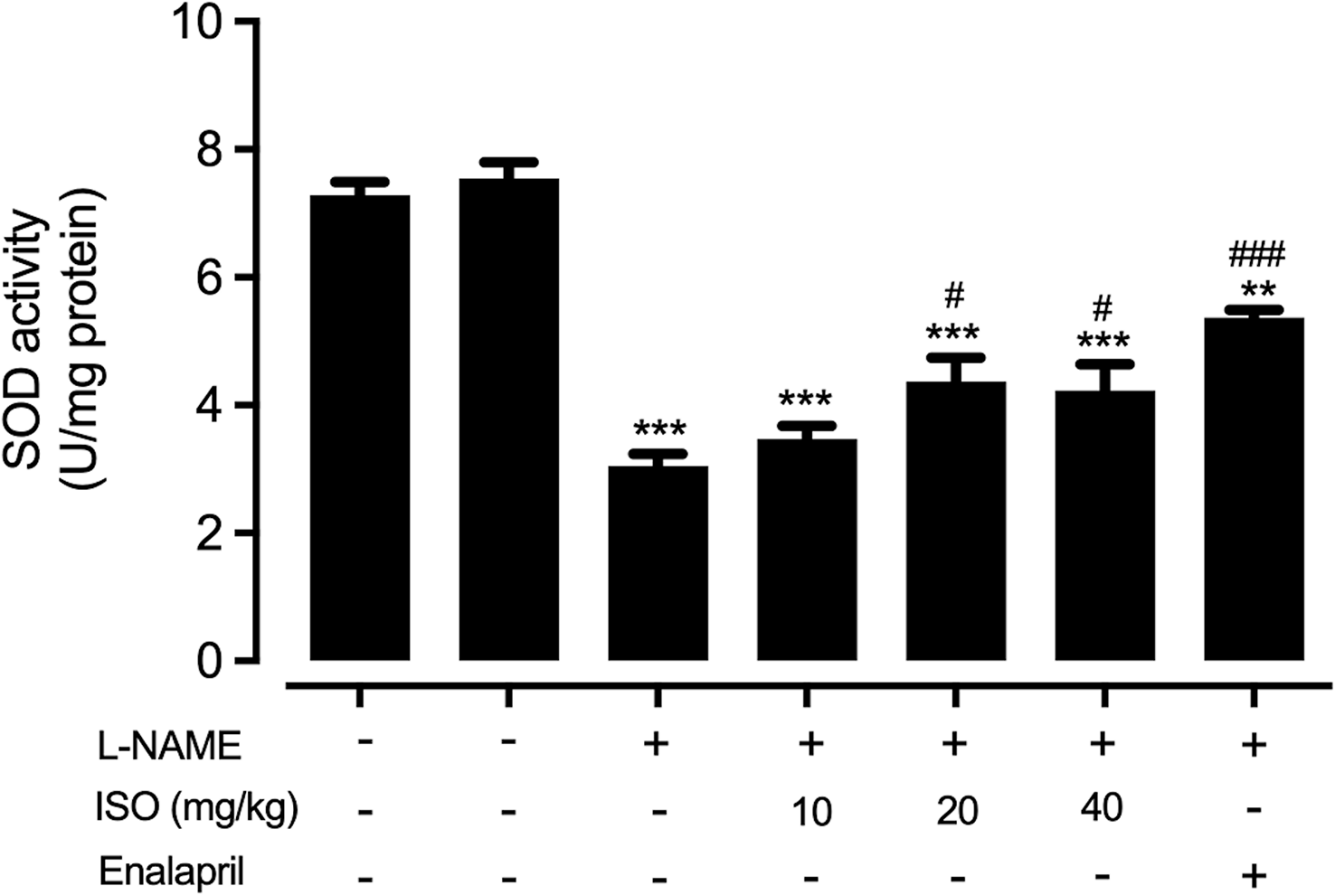
Effect of isosakuranetin (ISO) on superoxide dismutase (SOD) activity. The level of SOD activity was measured in different groups: control group, control group treated with ISO 40 mg/kg , hypertension group (L-NAME treated group), and hypertension groups treated with ISO at 10, 20, or 40 mg/kg or enalapril at 10 mg/kg. Data are presented as mean ± SEM. Statistical significance is indicated as **p < 0.01 and ***p < 0.001 vs. control group; ^#^*p* < 0.05 and ^###^*p* < 0.001 vs. hypertension group

To assess the antioxidative effect of isosakuranetin, the activity of SOD was evaluated from the isolated aorta. The results demonstrated that SOD activity was significantly reduced after L-NAME administration (*p* < 0.001). In contrast, treatment with 20 and 40 mg/kg of isosakuranetin significantly increased SOD activity (*p* < 0.05), showing effects comparable to those observed with 10 mg/kg of enalapril treatment (*p* < 0.001) when compared to the hypertension group as showed in Fig. 5.

## Discussion

The main findings of this study indicate that isosakuranetin increases NO levels and reduces oxidative stress, supporting the hypothesis that isosakuranetin has potential in ameliorating hypertension.

Endothelial cells, which line the vascular lumen, play a crucial role in regulating vascular tone by producing NO, prostaglandins, and other relaxing factors [37]. Endothelial dysfunction, characterized by NO depletion and increased ROS, impairs vasodilation, elevates oxidative stress, and contributes to hypertension and cardiovascular diseases [38, 39]. In this study, L-NAME, a well-known NOS inhibitor, was used to induce hypertension in animal models. The reduction in NO caused impaired vasodilation, increased vascular shear stress, and heightened oxidative stress [38]. Moreover, NO depletion upregulated NADPH oxidase, further increasing ROS production and exacerbating hypertension [40]. Consistent with previous studies, our results demonstrated that SBP was elevated while NO levels were reduced within four weeks of L-NAME administration. SBP was measured using the tail-cuff method, which provides reliable non-invasive assessment in conscious rats and serves as the primary endpoint in L-NAME hypertension studies. Although diastolic pressure was recorded, its high variability, characteristic of tail-cuff plethysmography, precluded inclusion in primary analyses [41]. This approach aligns with established L-NAME studies emphasizing SBP as the key functional outcome [42, 43].

While SBP was alter within 4 weeks, heart rate and heart weight were not changed. The absence of significant changes in heart weight aligns with previous findings that cardiac remodeling, such as hypertrophy, typically requires prolonged hypertension exposure beyond the treatment duration used in this study [44, 45]. Consistent with the lack of cardiac remodeling observed in this 6-week L-NAME model, histological examination of thoracic aorta via Hematoxylin and Eosin (H&E) staining (Supplementary Figure S1) revealed no significant vascular structural changes, such as medial thickening or fibrosis, across all groups.

Numerous studies have established that NO released from endothelial cells plays a pivotal role in regulating vascular tone and blood flow. Additionally, oxidative stress in the vasculature reduces NO bioactivity, thereby contributing to endothelial dysfunction and elevated blood pressure [39, 46]. Accordingly, improving endothelial function to enhance NO bioavailability has been a key therapeutic strategy for hypertension, and recent studies have highlighted the potential of natural supplements in this context [47, 48]. Maruyama and colleagues reported that isosakuranetin extracted from Brazilian green propolis reduced blood pressure and induced vasodilation in spontaneously hypertensive rats [34]. Nevertheless, further investigation is needed to fully elucidate the precise mechanisms by which isosakuranetin modulates blood pressure.

In this study, the observed restoration of NO levels by isosakuranetin, concurrent with blood pressure reduction following L-NAME-induced hypertension. This indicates enhanced endothelial nitric oxide synthase (eNOS) activity, similar to result observed in the enalapril-treated group. Enalapril, an angiotensin-converting enzyme inhibitor (ACEI), is commonly prescribed for hypertension and lowers blood pressure via the renin-angiotensin system (RAS). It has also been reported that ACEI treatment increases NO production in hypertensive patients [49, 50]. These results support the hypothesis that increased NO₂⁻ levels may reflect improved NO bioavailability, contributing to blood pressure reduction. However, future studies measuring eNOS protein/mRNA levels would provide direct confirmation of this mechanism .

In addition to endothelial dysfunction, oxidative stress is a key factor contributing to hypertension. Although ROS are generated physiologically during cellular metabolism, excessive accumulation is detrimental to vascular function. A primary enzymatic source of ROS in the vasculature is NADPH oxidase [51]. These ROS readily react with NO to form peroxynitrite (ONOO⁻), a reactive nitrogen species. This reaction reduces NO bioavailability and impairs vasodilation [52, 53]. Furthermore, ROS can increase intracellular calcium concentrations in vascular smooth muscle cells, promoting vasoconstriction [54]. Superoxide dismutase (SOD), an important endogenous antioxidant enzyme, catalyzes the dismutation of superoxide radicals into hydrogen peroxide and oxygen, thus serving as a critical defense against oxidative damage in the vasculature. By reducing superoxide levels, SOD helps preserve NO bioavailability and supports normal vasodilation [55, 56]. Thus, ROS contribute to hypertension by decreasing NO availability and indirectly promoting vasoconstriction. Consequently, antioxidant therapies that enhance SOD activity have gained interest as potential strategies to reduce oxidative stress and lower blood pressure.

Isosakuranetin treatment significantly enhanced SOD activity and reduced intracellular ROS levels in a dose-dependent manner, as measured by enzymatic assays and DCF-DA fluorescence, respectively. These markers provide established insight into enzymatic antioxidant defense and redox status in vascular models. This profile indicates that isosakuranetin strengthens endogenous defenses against superoxide-mediated damage, thereby preserving NO bioavailability and mitigating vasoconstriction in line with the observed blood pressure reduction. Notably, these antioxidant effects mirrored those of enalapril, aligning with mechanisms reported for ACEIs and flavonoids such as isorhamnetin, luteolin, quercetin, and kaempferol, which collectively reduce oxidative stress, vascular inflammation, and endothelial dysfunction [57–59], suggesting that enhancement of endogenous antioxidant enzyme systems is an effective mechanism for cardiovascular protection that can reduce atherosclerosis, vascular inflammation, oxidative stress, and endothelial dysfunction.

While SOD and ROS assessments elucidate key aspects of isosakuranetin’s redox modulation, they do not fully address lipid peroxidation (e.g., MDA) or non-enzymatic systems (e.g., GSH) [60]. Future studies incorporating these biomarkers would further delineate the compound’s comprehensive antioxidant profile and protective mechanisms.

These findings support that isosakuranetin’s antihypertensive effects occur primarily through rapid functional improvements in endothelial NO bioavailability and antioxidant defense, rather than structural remodeling, which typically requires longer hypertension duration.

## Conclusion

In conclusion, this study demonstrates that isosakuranetin effectively ameliorates hypertension by increasing nitric oxide bioavailability and reducing oxidative stress. The compound restores endothelial function impaired by L-NAME-induced NO depletion, leading to improved vasodilation and lowered blood pressure. Antioxidant potential of isosakuranetin, including the reduction of reactive oxygen species and enhancement of superoxide dismutase activity, further contributes to its cardiovascular protective effects. These findings support the potential of isosakuranetin as a natural therapeutic agent for hypertension, with efficacy comparable to enalapril. Future research should further elucidate the molecular mechanisms underlying its antihypertensive action and explore its clinical applications.

## Supplementary Information

Below is the link to the electronic supplementary material.

Supplementary Material 1

## Data Availability

No datasets were generated or analysed during the current study.

## Abbreviations

ACEI: Angiotensin-converting enzyme inhibitor
ANOVA: Analysis of variance
AT1: Angiotensin II type 1 receptor
C. odorata: Chromolaena odorata
DCF-DA: 2’,7’-dichlorofluorescein diacetate
DCFH-DA: 2’,7’-dichlorodihydrofluorescein diacetate
eNOS: Endothelial nitric oxide synthase
EtOAc: Ethyl acetate
L-NAME: L-N^G^-Nitroarginine methyl ester
MeOH: Methanol
NaNO₂: Sodium nitrite
NADPH: Nicotinamide adenine dinucleotide phosphate
NO: Nitric oxide
NO₂⁻: Nitrite ion
NOS: Nitric oxide synthase
ONOO⁻: Peroxynitrite
RAS: Renin–angiotensin system
ROS: Reactive oxygen species
SBP: Systolic blood pressure
SEM: Standard error of mean
SOD: Superoxide dismutase
TLC: Thin-layer chromatography
WHO: World Health Organization
