# Supplementary material for: Isosakuranetin ameliorates hypertension in rats induced by L-NAME
Source: J Pharm Health Care Sci. 2025 Dec 17;12:8. doi: 10.1186/s40780-025-00529-z (PMC12821859; doi:10.1186/s40780-025-00529-z)
Supplement: Supplementary file 1 — Supplementary Material 1 [file 40780_2025_529_MOESM1_ESM.pdf]

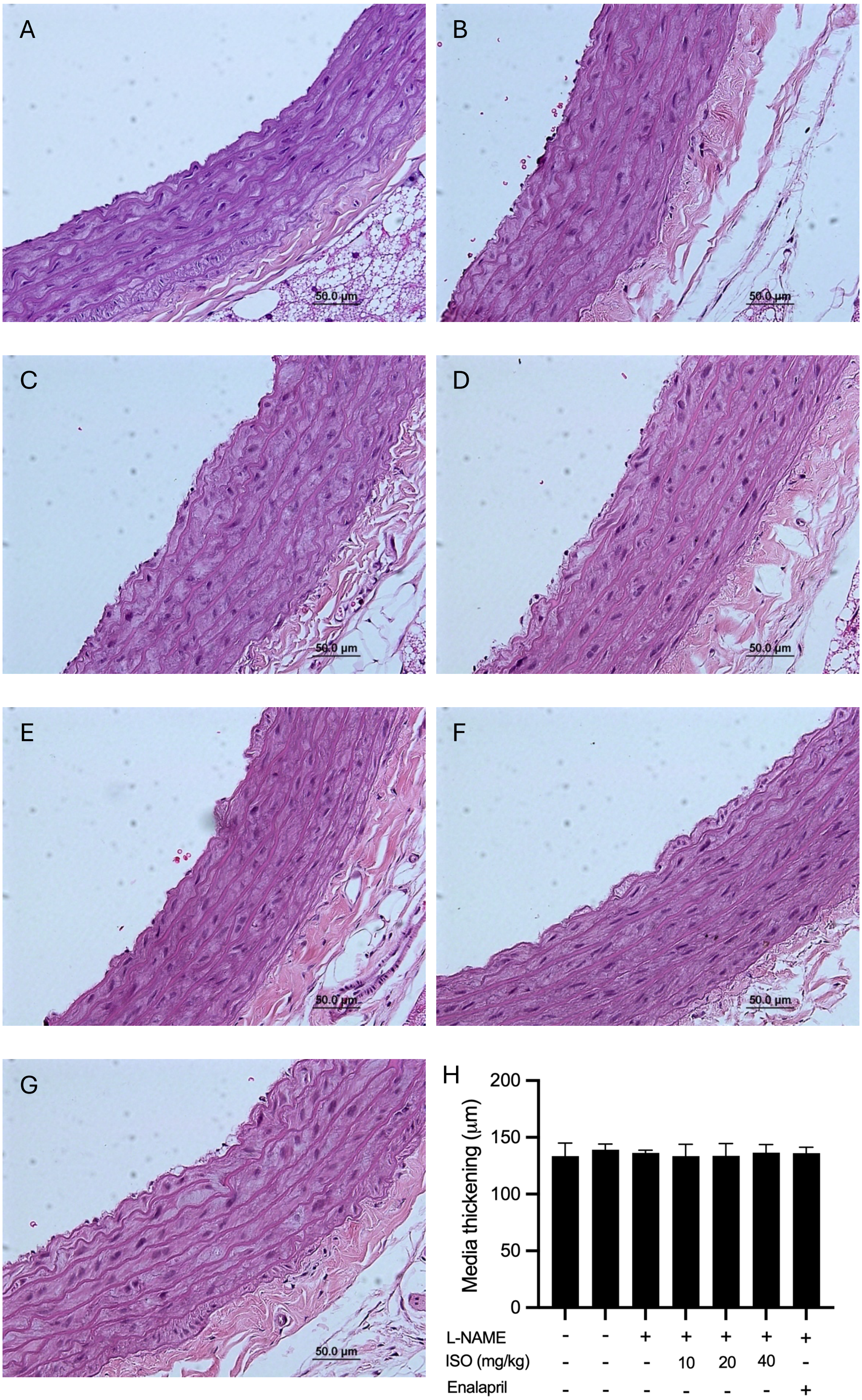

Supplementary Figure S1 Representative hematoxylin and eosin (H&E)-stained sections of thoracic aorta from rats in each experimental group: (A) Control, (B) L-NAME 40 mg/kg, (C) isosakuranetin (ISO) 40 mg/kg, (D) L-NAME+ISO 10 mg/kg, (E) L-NAME+ISO 20 mg/kg, (F) L-NAME+ISO 40 mg/kg, and (G) L-NAME+enalapril 10 mg/kg. The structure of the aortic wall is preserved without evidence of vascular remodeling, and medial thickness appears comparable among all groups. Quantitative analysis confirms that medial thickness does not differ significantly between groups (H); data are presented as mean ± SEM. Scale bar = 50.0 µm.
